# Supplementary material for: Structure of the NS2B-NS3 protease from Zika virus after self-cleavage
Source: Nat Commun. 2016 Nov 15;7:13410. doi: 10.1038/ncomms13410 (PMC5116066; doi:10.1038/ncomms13410)
Supplement: Supplementary Information — Supplementary Figures 1–10, Supplementary Table 1, Supplementary Note 1 and Supplementary References [file ncomms13410-s1.pdf]

Supplementary Figure 1. Amino acid sequence alignment of flaviviral NS2B-NS3 proteases. Residues conserved across flaviviruses are highlighted in yellow. eZiPro residues that were not resolved in the crystal structure are highlighted in gray. Residues at the crystallographic dimer interface are highlighted in cyan (Refer to **Supplementary Fig. 4**).

```
           45  47                                        83  87      96
eZiPro     TGKSVDMYIERAGDITWEKDAEVTGNSPRLDVALDESGDFSLVEEDGPPMRE-----------------------
WNV        SGKSTDMWIERTADITWESDAEITGSSERVDVRLDDDGNFQLMNDPGAPWKIWMLRMACLAISAYTPWAILPSVI
JEV        SGKATDMWLERAADISWEMDAAITGSSRRLDVKLDDDGDFHLIDDPGVPWKVWVLRMSCIGLAALTPWAIVPAAF
DENV4      SGSSADLSLEKAANVQWDEMADITGSSPIIEVKQDEDGSFSIRDVEETNMITLLVKLALITVSGLYPLAIPVTMT
DENV2      TGRSADLELERAADVKWEDQAEISGSSPILSITISEDGSMSIKNEEEEQTLTILIRTGLLVISGLFPVSIPITAA
DENV1      SGSSADLSLEKAAEVSWEEEAEHSGASHNILVEVQDDGTMKIKDEERDDTLTILLKATLLAVSGVYPLSIPATLF
DENV3      TGTSADLTVEKAADVTWEEEAEQTGVSHNLMITVDDDGTMRIKDDETENILTVLLKTALLIVSGIFPYSIPATLL
YFV        AGRVDGLELKKLGEVSWEEEAEISGSSARYDVALSEQGEFKLLSEEKVPWDQVVMTSLALVGAALHPFALLLVLA
TBEV       GTRKMQLVAEWSGCVEWYPELVNEGGEVSLRVRQDAMGNFHLTELEKEERMMAFWLIAGLAASAIHWSGILGVMG

                  126 130 1                 19                                 51
eZiPro     --------KTGKR-SGALWDVPAPKEVKKGE-TTDGVYRVMTRRL-LGSTQVGVGVMQEGVFHTMWHVTKGAALR
WNV        GFWIT--LQYTKR-GGVLWDTPSPKEYKKGD-TTTGVYRIMTRGL-LGSYQAGAGVMVEGVFHTLWHTTKGAALM
JEV        GYWLT--LKTTKR-GGVFWDTPSPKPCSKGD-TTTGVYRIMARGI-LGTYQAGVGVMYENVFHTLWHTTRGAAIM
DENV4      LWYMW--QVKTQR-SGALWDVPSPAATQKAA-LSEGVYRIMQRGL-FGKTQVGVGIHIEGVFHTMWHVTRGSVIC
DENV2      AWYLW--EVKKQR-AGVLWDVPSPPPMGKAE-LEDGAYRIKQKGI-LGYSQIGAGVYKEGTFHTMWHVTRGAVLM
DENV1      VWYFW--QKKKQR-SGVLWDTPSPPEVERAV-LDDGIYRIMQRGL-LGRSQVGVGVFQDGVFHTMWHVTRGAVLM
DENV3      VWHTW--QKQTQR-SGVLWDVPSPPETQKAE-LEEGVYRIKQQGI-FGKTQVGVGVQKEGVFHTMWHVTRGAVLT
YFV        GWLFH--VRGARRSGDVLWDIPTPKIIEECEHLEDGIYGIFQSTF-LGASQRGVGVAQGGVFHTMWHVTRGAFLV
TBEV       LWTLTEMLRSSRRSDLVFSGQGGRERGDRPFEVKDGVYRIFSPGLFWGQNQVGVGYGSKGVLHTMWHVTRGAALS

                          75                                                129 133
eZiPro     SGEGRLDPYWGDVKQDLVSYCGPWKLDAAWDGLSEVQLLAVPPGERAKNIQTLPGIFKTK-DGDIGAVALDYPAG
WNV        SGEGRLDPYWGSVKEDRLCYGGPWKLQHKWNGHDEVQMIVVEPGKNVKNVQTKPGVFKTP-EGEIGAVTLDYPTG
JEV        SGEGKLTPYWGSVKEDRIAYGGPWRFDRKWNGTDDVQVIVVEPGKAAVNIQTKPGVFRTP-FGEVGAVSLDYPRG
DENV4      HETGRLEPSWADVRNDMISYGGGWRLGDKWDKEEDVQVLAIEPGKNPKHVQTKPGLFKTL-TGEIGAVTLDFKPG
DENV2      HKGKRIEPSWADVKKDLISYGGGWKLEGEWKEGEEVQVLALEPGKNPRAVQTKPGLFKTN-AGTIGAVSLDFSPG
DENV1      YQGKRLEPSWASVKKDLISYGGGWRFQGSWNTGEEVQVIAVEPGKNPKNVQTAPGTFKTP-EGEVGAIALDFKPG
DENV3      HNGKRLEPNWASVKKDLISYGGGWRLSAQWQKGEEVQVIAVEPGKNPKNFQTMPGIFQTT-TGEIGAIALDFKPG
YFV        RNGKKLIPSWASVKEDLVAYGGSWKLEGRWDGEEEVQLIAAVPGKNVVNVQTKPSLFKVRNGGEIGAVALDYPSG
TBEV       IDDAVAGPYWADVREDVVCYGGAWSLEEKWKG-ETVQVHAFPPGRAHEVHQCQPGELILDTGRKLGAIPIDLVKG

           135             151       161             177
eZiPro     TSGSPILDKCGRVIGLYGNGVVIKNGSYVSAITQGKREEETPV--E
WNV        TSGSPIVDKNGDVIGLYGNGVIMPNGSYISAIVQGERMEEPAP--AG
JEV        TSGSPILDSNGDIIGLYGNGVELGDGSYVSAIVQGDRQEEPVP--EA
DENV4      TSGSPIINRKGKVIGLYGNGVVTKSGDYVSAITQAERIGE-PD--YE
DENV2      TSGSPIIDKKGKVVGLYGNGVVTRSGAYVSAIAQTEKSIE-DN--PE
DENV1      TSGSPIVNREGKIVGLYGNGVVTTSGTYVSAIAQAKASQEGPL--PE
DENV3      TSGSPIINREGKVVGLYGNGVVTKNGGYVSGIAQTNAEPDGPT--PE
YFV        TSGSPIVNRNGEVIGLYGNGILVGDNSFVSAISQTEVKEEGKE--EL
TBEV       TSGSPILNAQGVVVGLYGNGLKT-NETYVSSIAQGEAEKSRPNLPQA
```

Supplementary Figure 2. Recombinant ZIKV protease proteins. (A) Purified gZiPro, eZiPro and bZiPro are visualized on SDS-PAGE. Molecular weight markers were loaded for reference. gZiPro migrates at a MW of 25 kDa. eZiPro and bZiPro migrated between reference MW 20 and 15 kDa representing NS3 protease domain after cleavage of the NS2B-3 junction d. **(B)** Gel filtration profiles of gZiPro, eZiPro and bZiPro. All three proteins showed very similar gel filtration profiles. First two peaks represent aggregated proteins and gZiPro tends to aggregate more than eZiPro and bZiPro. Purified proteins were subjected to molecular weight determination using matrix assisted laser desorption ionization. The results of MW determinations are shown in **(C)** gZiPro **(D)** eZiPro and **(E)** bZiPro respectively. Peaks corresponding to NS2B cofactor residues, NS3 protease and uncleaved NS2B-NS3 ZIKV protease are indicated along with the determined molecular weights and expected molecular weights in parentheses.

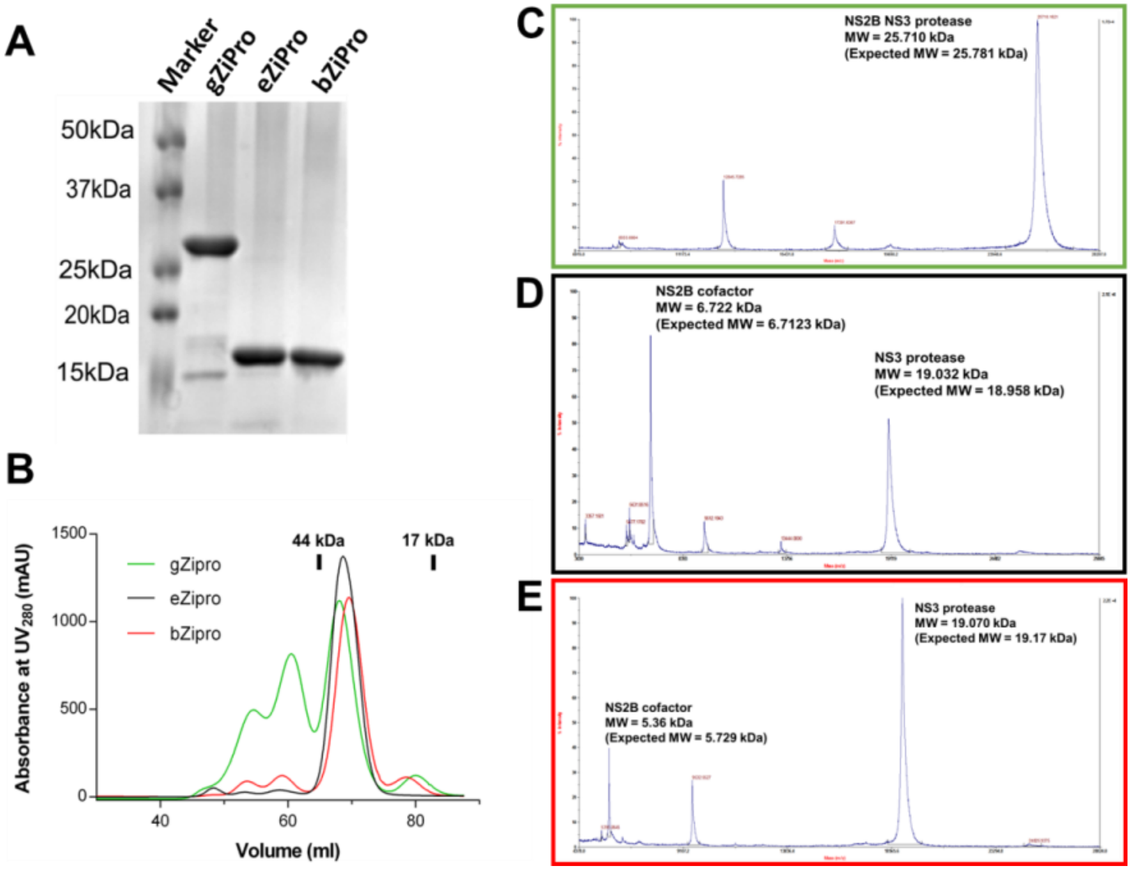

Supplementary Figure 3. Structure comparison of the NS2B-NS3 protease inhibitor complexes across flaviviruses. NS2B and NS3 of ZIKV are colored magenta and yellow. NS2B and NS3 of DENV and WNV are colored green and cyan. The inhibitors are colored in red. Refer to **Supplementary Table 1.**

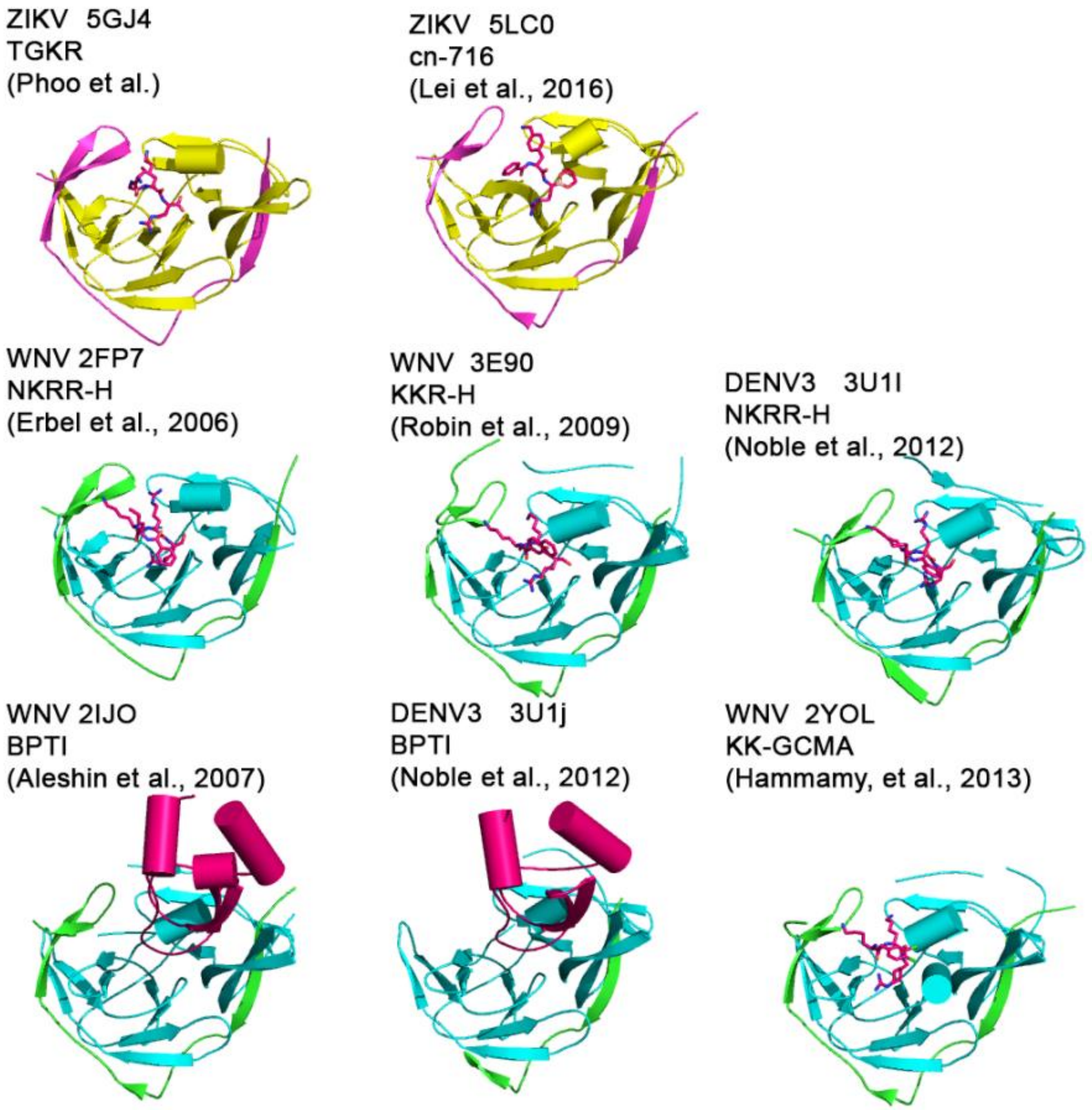

Supplementary Figure 4. Non-crystallographic dimer formed by eZiPro in the crystal (A) the assymetric unit contains four NS2B-NS3 molecules arranged as two dimers of NS2B-NS3 heterodimers each bound to one "TGKR" peptide. **(B)** Close-up view of one C:D -G:H ZIKV protease dimer generated by a non-crystallographic 2 fold symmetry axis. The relative orientation of the protease to the ER membrane is indicated. Arrows indicate the NS2B membrane association. **(C)** Close-up view of the dimeric interface interactions between the two eZiPro molecules, G:H and C:D. The NS3 protease is colored in orange and yellow, NS2B cofactor is colored in blue and magenta respectively. "TGKR" peptides are shown as sticks. Hydrogen bonds are represented by dashed lines. Residues belonging to the NS2B cofactor are underlined. All interacting pairs are listed in the lower panel. ZIKV specific interaction pairs are highlighted in bold font and labeled with a "*". The Surface Area buried at the dimer interface is 1859.2 Å^2 (calculated using AREAIMOL program[1,2] from the CCP4 suite)[3,4].

The dimer formation observed in crystal structure might be relevant biologically, where two NS2B-NS3 proteases come together forming dimers on ER membrane with hydrophobic residues of NS3 (L30L31) pointing towards ER as well during cis-cleavage reactions of NS2B-3 and NS2A-NS2B junctions. They can also facilitate efficient *trans* cleavage at the other polyprotein junctions.

A

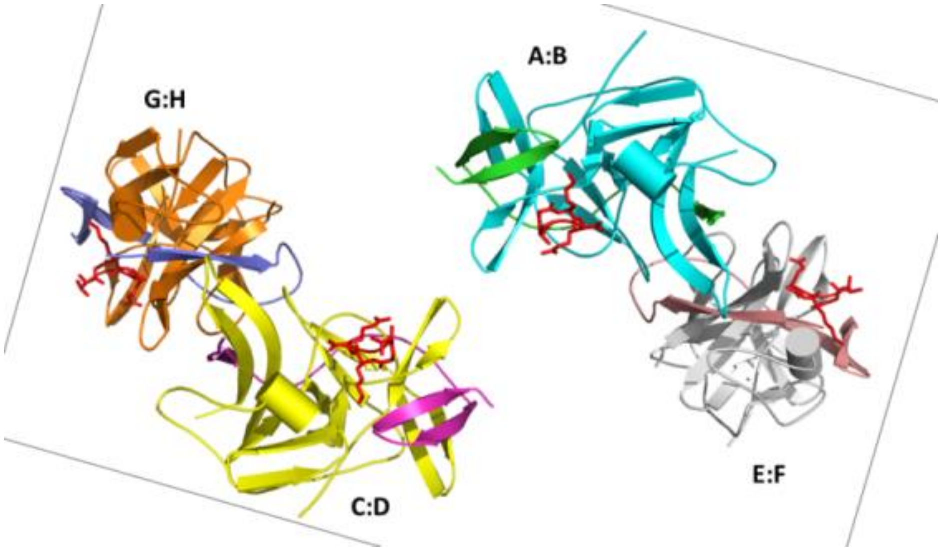

B

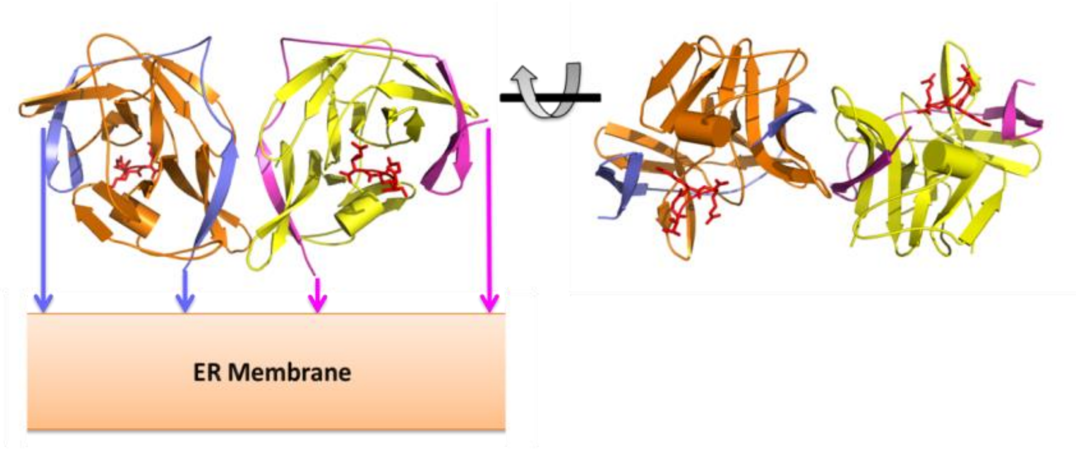

C

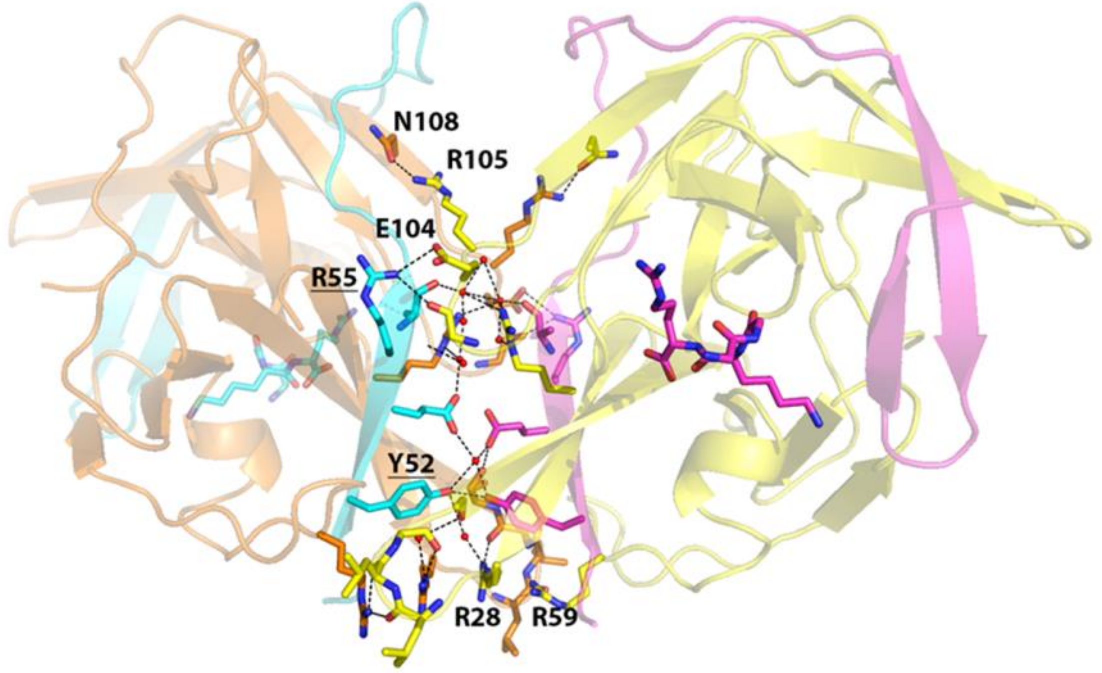

G NS2B	H NS3	C NS2B	D NS3
	***N108**		***R105**
	***R105**		***N108**
	***E104**	***R55**	
	G103	R55	
	L30		R59
	G32		R28
***Y52**		***Y52**	
	***R59/R28**		***R59/R28**
	R59		G32
	G32		R59
	R28		G32
R55			G103
***R55**			***E104**
	L30		R59

Supplementary Figure 5. Electron density map of TGKR peptide interacting with substrate site. NS2B is colored in magenta and NS3 in yellow. Catalytic triad residues are in orange. Solvent molecules are shown as red dots. $2mF_o$-DF_c map is contoured at 1.5σ in blue.

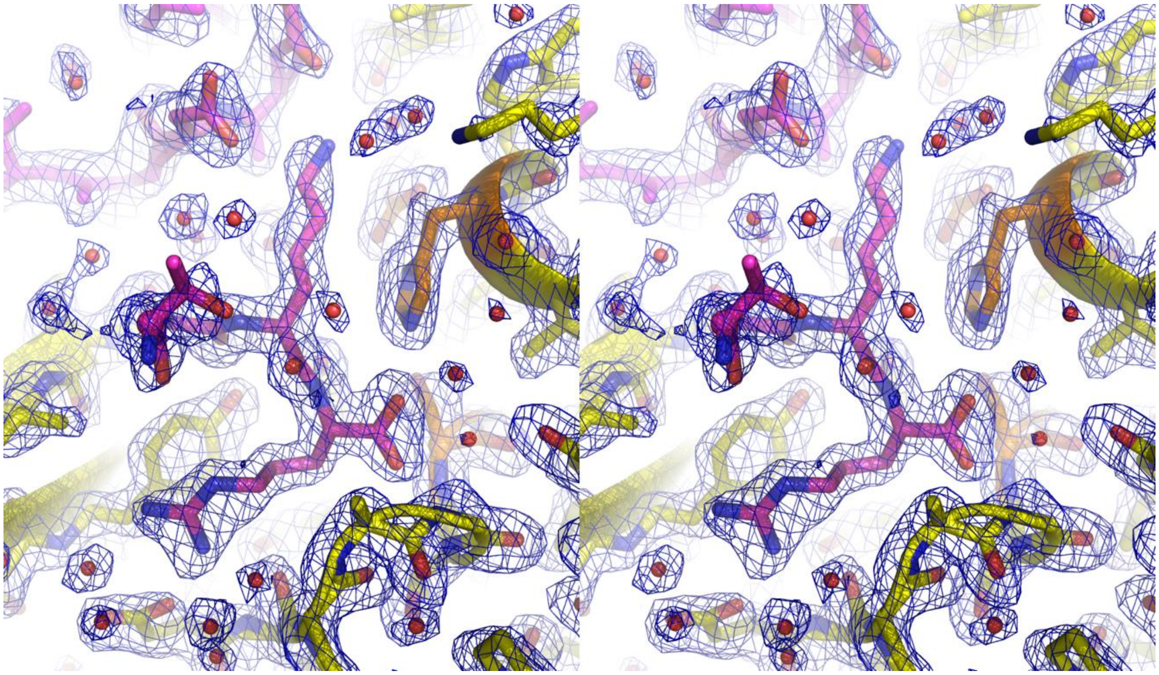

Supplementary Figure 6. Assignment of the ^{1}H-^{15}N-HSQC spectra of ZIKV proteases. The assignment was obtained using a sample in the NMR buffer that contained 20 mM HEPES, 150 mM NaCl, and 1 mM DTT. Cross peaks are labeled with the residue name and sequence number. An asterisk was added to distinguish residues belonging to the NS2B protein and highlighted in red. The C terminal region of NS2B including residues 76-86 of NS2B exhibited broadened peaks. Some cross peaks can be assigned as a second conformation of residues 76-86 of NS2B which are highlighted in green. These residues exhibited sharper peaks, indicating that the second conformation of NS2B 76-86 is very flexible. The assignment has been deposited into BMRB with access number 26873 .

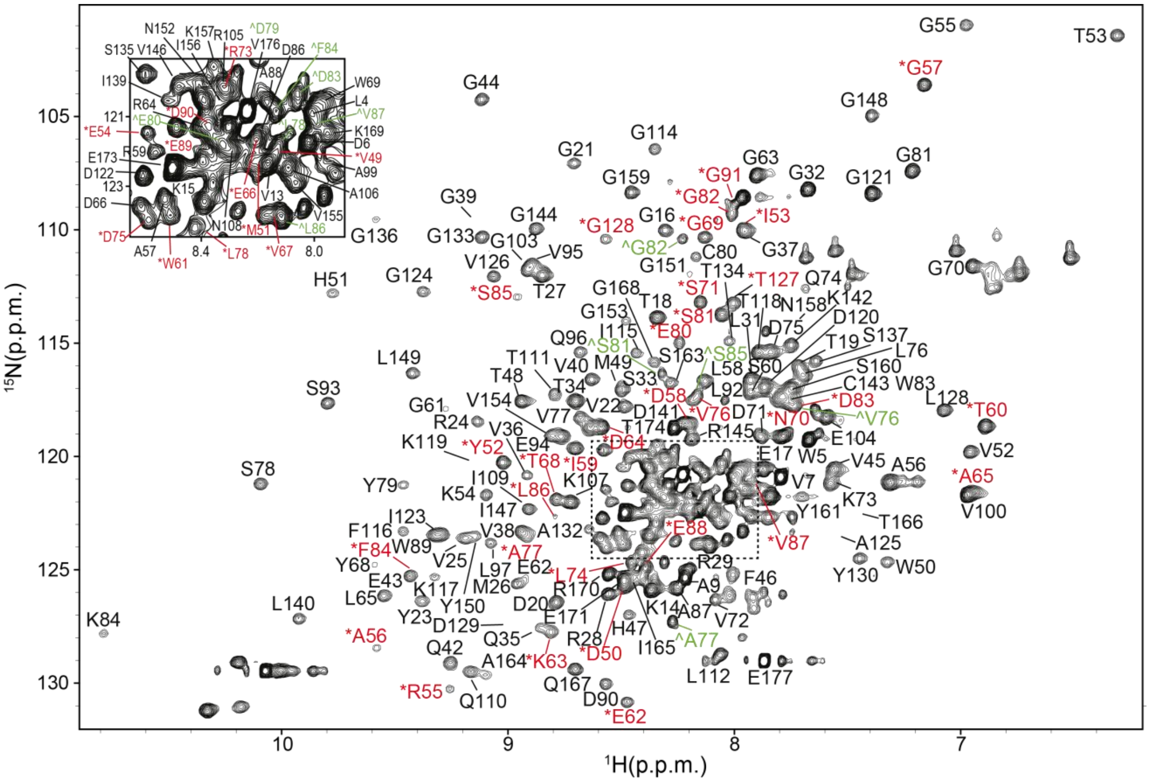

Supplementary Figure 7. Secondary structural analysis of eZiPro at pH7.3 and 37 °C. (A) The secondary structures of eZiPro in solution was obtained based on the assigned chemical shifts using TALOSN. Residues having helical and strand structures are shown in purple and black, respectively. Secondary structural elements derived from X-ray structures are shown in boxes (helix) and arrows (strands), respectively. The assignments for T127-G128-K129-R130 peptide were not unambiguously assigned, which may be due to intermediate exchanges and signal overlaps. Residues 73-75 of NS2B tend to form a β-strand, consistent with the X-ray structure. Residues 85-87 of NS2B have a tendency to be a β-strand, but the possibility is low, which may be due to its dynamics. (B) Residue L74 of NS2B and K117 of NS3 has a close contact. The distance for the amide protons is 3.2 Å. (C) NOE observed between L74 of NS2B and K117 of NS3. Strip plot of NOESY-TROSY experiment is shown and NOEs between amide protons of L74 of NS2B and K117 of NS3 was observed, conforming that these two residues are in close contact and the structure is in the closed conformation.

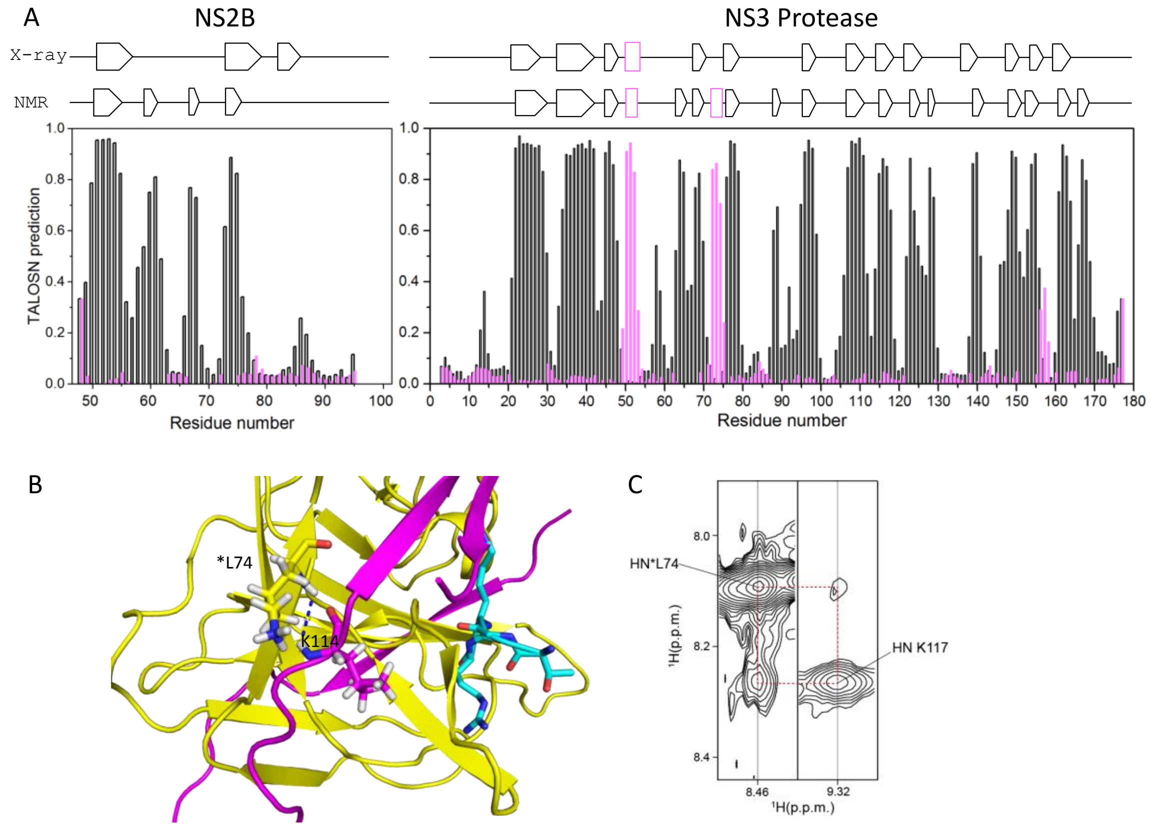

Supplementary Figure 8. Thermal shift assay of the three ZIKV protease constructs. Thermostabilities of the three proteases were analyzed. The Tm value for each construct was generated from the heat induced melting curves. Protein concentrations: 15 µM; Dye: 20x; White plate Protein storage and dilution buffer: 20 mM Hepes, pH 7.3, 180 mM NaCl, 1 mM DTT. The assay was conducted using the method descripted previously[5,6]**.**

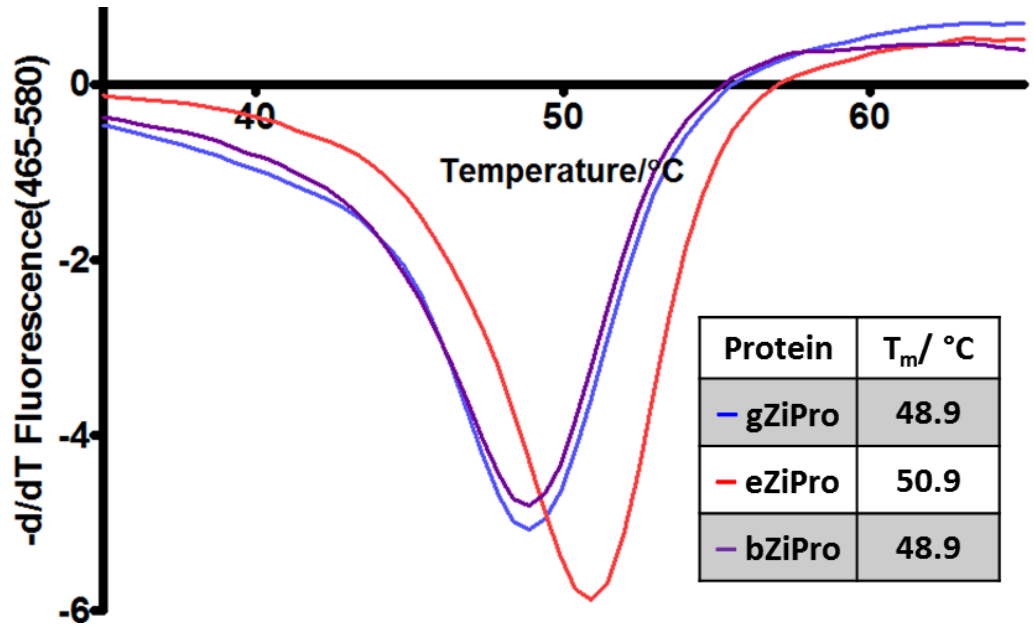

Supplementary Figure 9. ^{1}H-^{15}N-HSQC spectra of the purified proteases. A. ^{1}H-^{15}N-HSQC spectra of eZiPro (left), gZiPro (middle), and bZiPro (right) proteases. The NMR spectra were collected at 37 °C (310K). The samples were prepared in a buffer containing 20 mM sodium phosphate, pH 6.5, 150 mM NaCl, and 1 mM DTT.

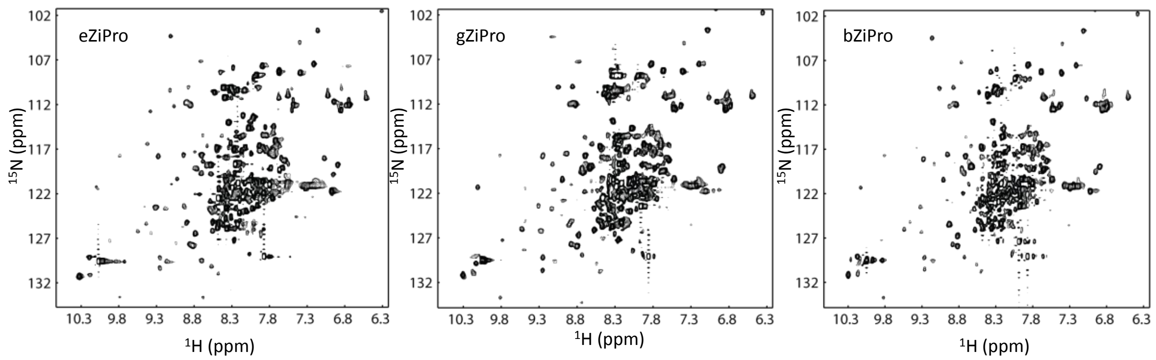

Supplementary Figure 10. Residues affected by TGKR binding. The residues exhibited different cross peaks in the NMR spectra (Fig. 2B) were mapped to the structure. NS3 protease and NS2B are presented as cartoon and ribbon modes, respectively. Residues exhibited line broadening in the ^{1}H-^{15}N-HSQC of bZiPro are shown as sphere and residues exhibited chemical shift difference between the two constructs in the ^{1}H-^{15}N-HSQC spectra are highlighted in green.

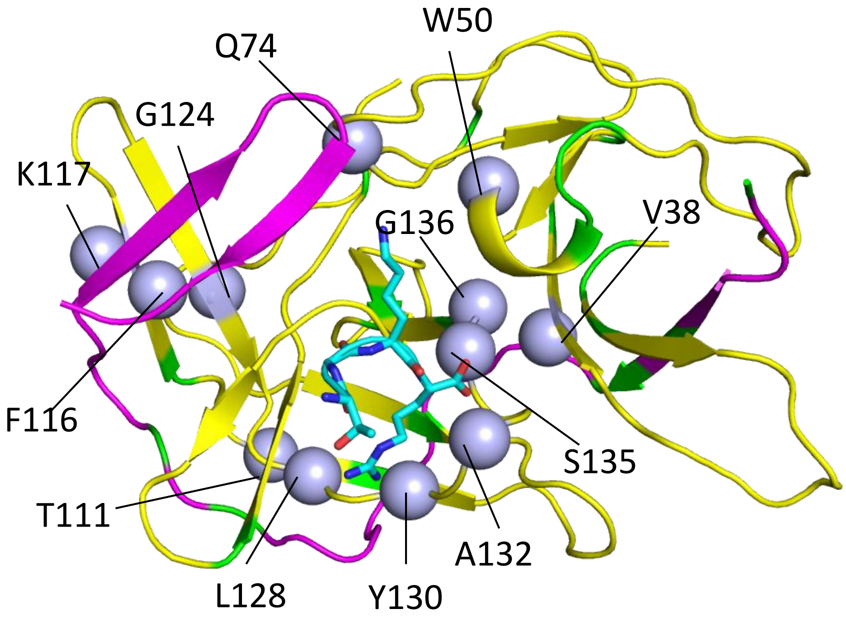

Supplementary Table 1 List of flaviviral NS2B-NS3 proteases in complex with inhibitors. Refer to **Supplementary Fig. 3**.

Virus	PDB	RMSD (Å)	Ligands	Reference
ZIKV	5GJ4	**0**	Thr-Gly-Lys-Arg	This study
	5LC0	**0.466**	Cn-716	[7]
DENV3	3U1I	**0.599**	Bz-Nle-Lys-Arg-Arg-H	[8]
	3U1J	**0.462**	BPTI	
WNV	2YOL	**0.567**	DCPA-Lys-Lys-GCMA	[9]
	3E90	**0.469**	NPAH-Lys-Lys-Arg-H	[10]
	2FP7	**0.417**	Bz-Nle-Lys-Arg-Arg-H	[11]
	2IJO	**0.451**	BPTI	[12]

Supplementary Note 1

Protease activity

Bessaud et al. reported the enzymatic activity of recombinant ZIKV protease on Boc-Gly-Arg-Arg-AMC substrates. The enzymatic activity of ZIKV protease against Boc-GRR-AMC substrates were found to have a much lower k_{cat} (0.075 s^{-1}) but a higher K_m value of 417 µM[13]. Li et al reported that differences in substrates used and various serotypes of dengue could yield large differences in enzyme kinetics[14]. Hence, here the observed difference between our ZIKV constructs and those by Bessaud et al could be either due to the use of different substrates, due to differences in protein constructs or both. Previous enzymatic studies of NS2B-NS3pro from various flaviviruses such as DENV1-4, JEV and YFV have been performed on the same substrate [14-18]. All ZIKV proteases showed higher catalytic rates compared to both JEV and YFV NS2B-NS3pro (k_{cat} = 0.01 s^{-1} and 0.11 s^{-1} respectively) and similar rates to DENV4 NS2B-NS3pro (kcat = 2.9 s^{-1}) on the same substrates, while substrate binding affinity of these protease were found to be similar to NS2B-3 proteases from DENV1-4 (K_m ranging from 6.2-12 µM reported by Li et al.[14] and from YFV (K_m = 14 µM), while JEV NS2B-NS3pro has higher K_m of 20 µM [15]. Sequence variations may thus contribute to the enhanced protease activity of ZIKV and its pathogenesis (**Supplementary Figure 1**).

Supplementary References

1 Saff, E. B. & Kuijlaars, A. B. j. *The Mathematical Intelligencer* **19**, 5-11 (1997).

2 Lee, B. & Richards, F. M. The interpretation of protein structures: estimation of static accessibility. *Journal of molecular biology* **55**, 379-400 (1971).

3 Potterton, E., Briggs, P., Turkenburg, M. & Dodson, E. A graphical user interface to the CCP4 program suite. *Acta crystallographica. Section D, Biological crystallography* **59**, 1131-1137 (2003).

4 Winn, M. D. *et al.* Overview of the CCP4 suite and current developments. *Acta crystallographica. Section D, Biological crystallography* **67**, 235-242, doi:10.1107/S0907444910045749 (2011).

5 Kang, C. *et al.* Biophysical Studies of Bacterial Topoisomerases Substantiate Their Binding Modes to an Inhibitor. *Biophys J* **109**, 1969-1977, doi:10.1016/j.bpj.2015.10.001
S0006-3495(15)01001-2 [pii] (2015).

6 Gayen, S. *et al.* An NMR study of the N-terminal domain of wild-type hERG and a T65P trafficking deficient hERG mutant. *Proteins* **79**, 2557-2565, doi:10.1002/prot.23089 (2011).

7 Lei, J. *et al.* Crystal structure of Zika virus NS2B-NS3 protease in complex with a boronate inhibitor. *Science*, doi:10.1126/science.aag2419 (2016).

8 Noble, C. G., Seh, C. C., Chao, A. T. & Shi, P. Y. Ligand-bound structures of the dengue virus protease reveal the active conformation. *J Virol* **86**, 438-446, doi:10.1128/jvi.06225-11 (2012).

9 Hammamy, M. Z., Haase, C., Hammami, M., Hilgenfeld, R. & Steinmetzer, T. Development and characterization of new peptidomimetic inhibitors of the West Nile virus NS2B-NS3 protease. *ChemMedChem* **8**, 231-241, doi:10.1002/cmdc.201200497 (2013).

10 Robin, G. *et al.* Structure of West Nile virus NS3 protease: ligand stabilization of the catalytic conformation. *J Mol Biol* **385**, 1568-1577, doi:10.1016/j.jmb.2008.11.026 (2009).

11 Erbel, P. *et al.* Structural basis for the activation of flaviviral NS3 proteases from dengue and West Nile virus. *Nat Struct Mol Biol* **13**, 372-373, doi:nsmb1073 [pii]
10.1038/nsmb1073 (2006).

12 Aleshin, A. E., Shiryaev, S. A., Strongin, A. Y. & Liddington, R. C. Structural evidence for regulation and specificity of flaviviral proteases and evolution of the Flaviviridae fold. *Protein science : a publication of the Protein Society* **16**, 795-806, doi:ps.072753207 [pii]
10.1110/ps.072753207 (2007).

13 Bessaud, M. *et al.* Functional characterization of the NS2B/NS3 protease complex from seven viruses belonging to different groups inside the genus Flavivirus. *Virus Res* **120**, 79-90, doi:S0168-1702(06)00040-2 [pii]
10.1016/j.virusres.2006.01.021 (2006).

14 Li, J. *et al.* Functional profiling of recombinant NS3 proteases from all four serotypes of dengue virus using tetrapeptide and octapeptide substrate libraries. *J Biol Chem* **280**, 28766-28774, doi:10.1074/jbc.M500588200 (2005).

15 Lohr, K. *et al.* Yellow fever virus NS3 protease: peptide-inhibition studies. *J Gen Virol* **88**, 2223-2227, doi:10.1099/vir.0.82735-0 (2007).
16 Mueller, N. H., Yon, C., Ganesh, V. K. & Padmanabhan, R. Characterization of the West Nile virus protease substrate specificity and inhibitors. *Int J Biochem Cell Biol* **39**, 606-614, doi:S1357-2725(06)00305-0 [pii]
10.1016/j.biocel.2006.10.025 (2007).
17 Yusof, R., Clum, S., Wetzel, M., Murthy, H. M. & Padmanabhan, R. Purified NS2B/NS3 serine protease of dengue virus type 2 exhibits cofactor NS2B dependence for cleavage of substrates with dibasic amino acids in vitro. *J Biol Chem* **275**, 9963-9969 (2000).
18 Junaid, M. *et al.* Enzymatic analysis of recombinant Japanese encephalitis virus NS2B(H)-NS3pro protease with fluorogenic model peptide substrates. *PloS one* **7**, e36872, doi:10.1371/journal.pone.0036872 (2012).
